# cTFbase: a database for comparative genomics of transcription factors in cyanobacteria

**DOI:** 10.1186/1471-2164-8-104

**Published:** 2007-04-18

**Authors:** Jinyu Wu, Fangqing Zhao, Shengqin Wang, Gang Deng, Junrong Wang, Jie Bai, Jianxin Lu, Jia Qu, Qiyu Bao

**Affiliations:** 1 Institute of Biomedical Informatics/Zhejiang Provincial Key Laboratory of Medical Genetics, Wenzhou Medical College, Wenzhou 325000, China; 2 Institute of Oceanology, Chinese Academy of Sciences, Qingdao 266071, China; 3 Department of Biochemistry and Molecular Biology, Pennsylvania State University, Pennsylvania 16802, USA

## Abstract

**Background:**

Comprehensive identification and classification of the transcription factors (TFs) in a given genome is an important aspect in understanding transcriptional regulatory networks of a specific organism. Cyanobacteria are an ancient group of gram-negative bacteria with strong variation in genome size ranging from about 1.6 to 9.1 Mb and little is known about their TF repertoires. Therefore, we constructed the cTFbase database to classify and analyze all the putative TFs in cyanobacterial genomes, followed by genome-wide comparative analysis.

**Description:**

In the current release, cTFbase contains 1288 putative TFs identified from 21 fully sequenced cyanobacterial genomes. Through its user-friendly interactive interface, users can employ various criteria to retrieve all TF sequences and their detailed annotation information, including sequence features, domain architecture and sequence similarity against the linked databases. Furthermore, cTFbase provides phylogenetic trees of individual TF family, multiple sequence alignments of the DNA-binding domains and ortholog identification from any selected genomes. Comparative analysis revealed great variability of the TF sequences in cyanobacterial genomes. The high variance on the gene number and domain organization would be related to their diverse biological functions and their adaptation to various environmental conditions.

**Conclusion:**

cTFbase provides a centralized warehouse for comparative analysis of putative TFs in cyanobacterial genomes. The availability of such an extensive database would be of great interest for the community of researchers working on TFs or transcriptional regulatory networks in cyanobacteria. cTFbase can be freely accessible at  and will be continuously updated when the newly sequenced cyanobacterial genomes are available.

## Background

Deciphering and reconstructing gene transcriptional regulatory networks are important for better understanding the fundamental cellular processes, such as cell division, growth control and gene expression by which cells can adapt to environment more effectively [1]. The most basic components of transcriptional regulatory networks are the transcription factors (TFs), TF binding sites at upstream and downstream of the target genes and the target genes. Among them, TFs play the crucial role by enhancing or inhibiting the target gene expression by means of binding to the promoter sequences. Studies on TFs are of extreme significance and can glean more information about the mechanism of transcriptional regulatory networks. Genome-wide analysis of completely sequenced genomes have revealed that TFs account for a large proportion of all encoded proteins [2-4]. *Escherichia coli *was one of the best-studied organisms and was revealed to have more than 271 TFs in its whole genome. Despite the divergent domain organizations and sequence identities, the TFs characterized to share a significant degree of structural similarity of the DNA-binding domain (DBD), which binds to the specific DNA region [5]. TFs can be classified into several families based on structure difference of DBD which include helix-turn-helix motif, Zinc fingers, Leucine zippers, and Basic-helix-loop-helix, etc. Helix-turn-helix motif is the most common structure of DBD in prokaryotes [3,4,6]. Cyanobacteria are an ancient group of gram-negative bacteria, which exhibit extraordinary diversity in physiological properties, ecological niches and morphology [7]. They survive in different environments, such as fresh and marine waters and extreme conditions. Different member of cyanobacteria shows a remarkable size variation ranging from about 1.6 to 9.1 Mb. For example, *Prochlorococcus sp*. 1986 is only 1.75 Mb in genome size which is supposed to be one of the smallest genomes and most compact oxyphototrophic organisms discovered to date [8]. *Nostoc punctiforme *has a large genome size and complicated ecological niches which may suggest a relatively sophisticated organization of this species [9]. Currently, 21 cyanobacterial genomes have been fully sequenced, representing a wide range of species from unicellular to filamentous ones. In addition, more than 20 members of cyanobacteria are in the process of in-finishing or being sequenced [10,11]. Such data resources undoubtedly provide an opportunity for genome-wide analysis of TFs in cyanobacteria.

To date, only a few numbers of TFs in cyanobacteria have been studied in detail. However, those studied provide useful insights into the crucial roles of TFs in biology, and further into their functions. NtcA, one of the extensively studied TF that mediates global nitrogen control and regulates many genes involved in nitrogen assimilation, was identified in all cyanobacteria [12]. FUR could regulate iron assimilation and storage, and modulate the expression of genes involved in the response to different environmental stresses [13]. NtcB, another member of TFs, was identified to activate nitrate assimilation [14]. In order to make all the putative TFs in cyanobacteria available to the scientific community and fill in the gap without online databases, we present cTFbase, which will be a valuable resource for further research of TFs and transcriptional regulatory networks in cyanobacteria. Whole genome comparative analysis revealed great variability of the TFs in cyanobacterial genomes. The high variance on the gene number and domain organization would be related to their diverse biological functions and their adaptation to various environmental conditions.

## Construction and content

### Collection of TFs

**Figure 1:**
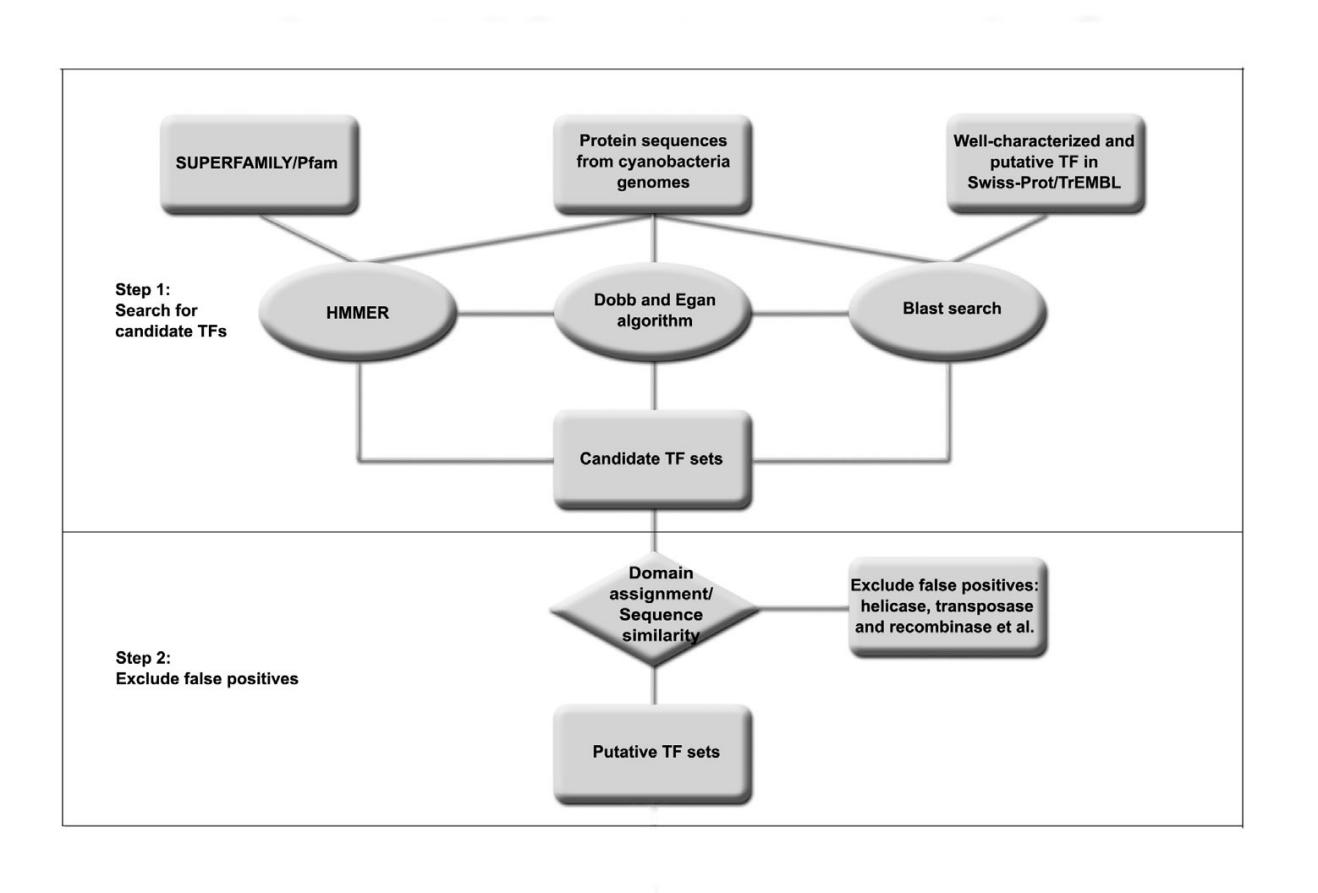
Flow-chart of method used to identify TFs.

The amino acid sequences of the protein-encoding genes of 21 cyanobacterial genomes were retrieved from the IMG database (version 2.0). A number of bioinformatics tools were used to identify the TFs (Fig. 1). The identification process included searching candidate TFs and their verification. In the first step, three different methods were performed to collect all possible TFs: (1) DBD assignment by the SUPERFAMILY database (release 1.69) and Pfam database (version 21.0)[15,16]. Varieties of DBDs were extracted from both two databases. The hmmpfam program in the HMMER package [17] was used to predict the domain(s) of a query protein sequence and those sequences with an E-value below 0.01 were selected; (2) BLAST search. An initial set of well characterized putative TFs was obtained from the Swiss-prot/TrEMBL databases (release 10.0) by a keyword search on the ExPASy molecular Biology server [18]. BLASTP searches were performed using this set as a query with E-value < 1E-10. The searches were iterated until no newly retrieved sequences belonged to TF; (3) Scanning for helix-turn-helix motif. This process is completed by the program Helixturnhelix implemented in EMBOSS with the default parameters [19]. Results obtained by above three methods were combined, and duplicate records, which were identified on the basis of locus tags, were removed. In the second step all the candidate TFs were manually checked and the false-positives were removed based on domain assignment and sequence similarity against main databases, such as Swiss-prot (release 52.0) and Refseq (release 22). This work focuses mainly on specific TFs that bind various regulatory elements. Hence, some general TFs, such as sigma factor, which binds the core promoter of a gene, were also excluded. Finally, we identified 1288 putative TFs from 21 cyanobacterial genomes, among which 404 genes have not been annotated as transcriptional factor (including neither shown the function of regulation, nor possessing the helix-turn-helix motif) in its original annotation in the IMG database. Some transcription factor databases such as DBD also identified TFs from cyanobacterial genomes [20]. However, we found more TFs (1288 items) from 21 cyanobacterial genomes than DBD database (version 2.0, 947 items) that was based on 17 genomes. Even if we compared the TFs from these 17 shared genomes, there are a number of discrepancies between them. First, DBD method [20] aimed to collect all the proteins containing DNA binding domain, which are sometimes not necessarily involved in the regulation of gene expression, such as transposase, integrase, recombinase, or methyltransferase. We found 191 curated TFs from the shared 17 genomes that are not present in DBD database, and meanwhile DBD also identified 128 putative TFs, which were considered as to be possible false positives in our method. Detailed information can be found from the *introduction *section of website. Second, DBD database was based on domain assignments from the SUPERFAMILY and PFAM HMM libraries, and predicted TFs from all eubacteria (including cyanobacteria) and eukaryotic organisms using the same benchmark. Here, several independent methods as described above were applied to identify TFs from cyanobacterial genomes, and special attention was paid to those hypothetical proteins and cyanobacterial-specific proteins.

### Implementation and web interface

The popular MySQL backend was used as the database machine to store the results. Web interfaces for database browsing and the browse result pages were developed using PHP scripts. Through the cTFbase web system, the following main functionalities are implemented in the current release:

(1) To browse domain architectures of TFs. The repertoire of TFs can be browsed and the domain architectures can be viewed from a selected genome. It can also show the special TF families and their related domain architectures in one or all the cyanobacterial genomes;

(2) To identify the orthologs from the selected genomes. The results of orthology relationships from multiple species was previously performed using the program OrthoMCL [21];

(3) To browse the phylogeny of individual TF family. Using the neighbor joining method, the phylogenetic tree for each of the TF families was constructed based on the whole TF sequences. The reasons for using the whole sequences of TFs instead of their DBD motifs to perform phylogenetic analysis was that the sequence of DBD motif in specific family is well conserved and the length is quite short, which may lead to very few deep nodes supported by high bootstrap values;

(4) To search the database via protein ID, species or family;

(5) To perform a BLAST-based sequence similarity search. Users can search their target sequences or identify homologs in the database;

(6) To perform sequence alignment. The sequence alignment tool, MUSCLE [22], was implemented to enable users to align amino acid sequences of DBDs within the specific families;

(7) To link some useful references, including literatures and databases;

(8) To download one (or all) specific TF sequence(s), including proteins and/or their corresponding DNA sequences, and phylogenetic trees in phylip format.

Furthermore, each entry provides the sequence itself and detailed annotations, including basic information, domain architecture assigned by Pfam database (version 21.0) and SUPERFAMILY database (release 1.69) and sequence similarity against major databases (PDB collected by 11-March-2007, Swiss-prot release 52.0, Refseq release 22 and DBD version 2.0). Through links to inner section and other databases, it would be a platform in which information on putative TFs in cyanobacteria has highly integrated and will be a centralized warehouse for the comparative genomic analysis. We expect that this database will help to further understand the transcriptional regulatory networks of microbiology.

## Utility and discussion

### Comparison of TFs among different species of cyanobacteria

**Figure 2:**
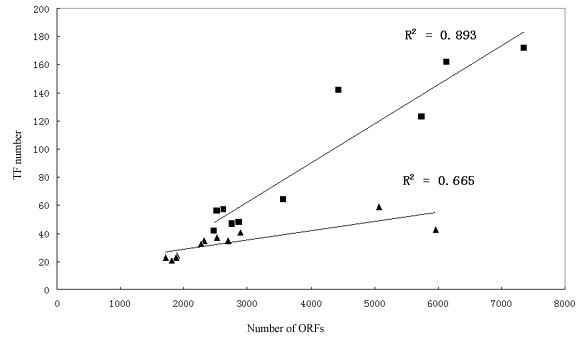
Relationship between the number of TFs and the total number of ORFs in 21 cyanobacterial genomes. The total number of ORFs in each genome was plotted as a function against the number of TFs. Triangle indicates marine cyanobacteria, while square indicates fresh or soil cyanobacteria.

Different from other prokaryotes, cyanobacteria exhibit extraordinary diversities in physiology, ecological niches and morphology. Genome-wide analysis of TFs undoubtedly helps to understand the relationship between cyanobacterial TF composition and their environmental adaptation. Here, we found that the cyanobacteria living in fresh water or soil has a larger amount of putative TFs comparing to those living in marine water (The distribution of TFs in these 21 cyanobacterial genomes can be seen in the *statistics *section of website). Given that the genome size of the fresh water or soil cyanobacteria is much larger than those of the marine species and it is well known that growth in bacterial genome size is accompanied with the accumulation of paralogous protein families [23], we then calculated the relative number of TFs in cyanobacteria. The results demonstrate that the relative number of TFs in fresh water or soil cyanobacteria is still significantly higher than that in marine cyanobacteria (Fig. 2). Similar results were also found in the cyanobacterial signal transduction and restriction-modification system [24,25]. Fresh water or soil is considered as a less stable environment than a marine ecosystem, with drastic changes of temperature and light, abundant but inconstant nutrient resources and more potential hazards. Therefore, the genome of fresh water or soil species encodes multiple capabilities in order to survive in this unstable environment and to have a long-term selective advantage. TF should be one of such capabilities, because it is essential for organisms to control the expression of the target gene(s) to adapt for the environmental changes effectively. For example, *Nostoc punctiforme*, a filamentous nitrogen-fixing cyanobacterium, displays an extraordinarily wide range of developmental alternatives in vegetative cell, physiological properties, and ecological niches, including broad symbiotic competences with plants and fungi [9]. Within its genome, a total of 172 putative TFs were identified, representing the most abundant in all sequenced cyanobacterial genomes. On the other hand, *Prochlorococcus*, living in the oligotrophic open ocean and regarded as one of the most compact oxyphototrophic organisms [26], possesses a limited number of putative TFs (usually less than 30). It is assumed that the main driving force behind genome reduction within the *Prochlorococcus *is radiation, which has been the selective process favoring the adaptation of this organism to its environment [27,28].

**Figure 3:**
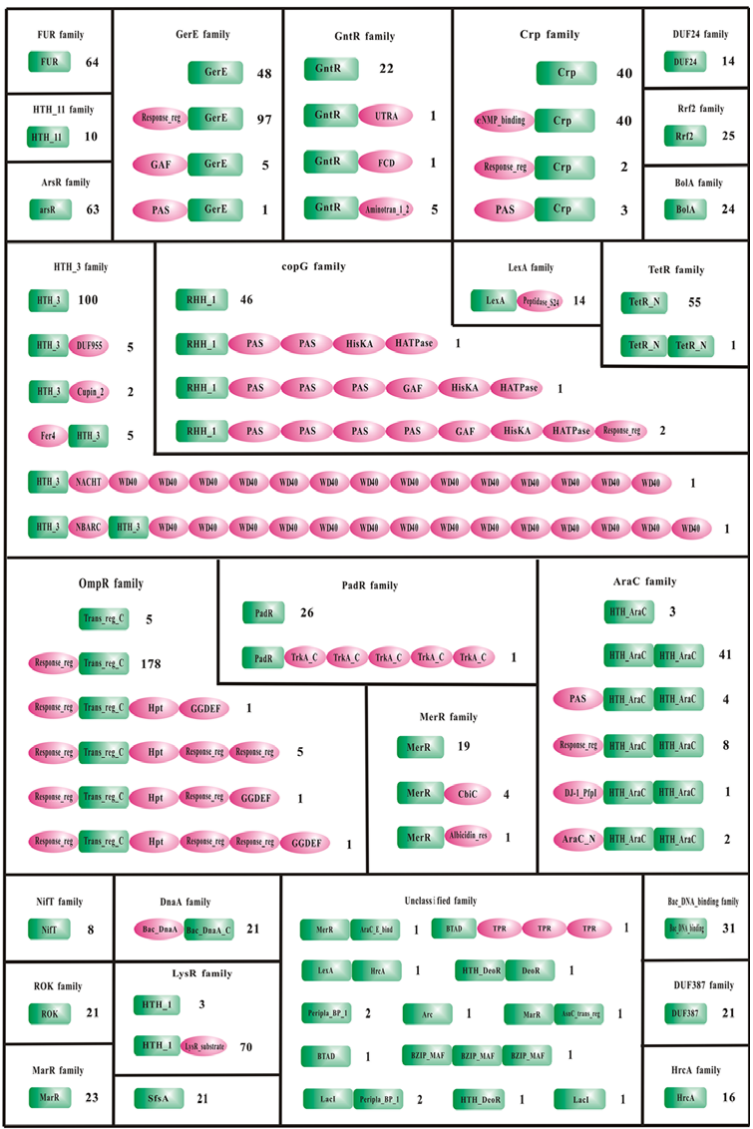
Domain architectures and classification scheme of TFs in the 21 cyanobacterial genomes. The methods classifying TFs into families and identifying the domains can be referred in *Section 2*. Green rectangles indicate the DBD domains, while red ellipses represent associated domains. The number on the right of the domain organization represents the number of sequences in the specific family.

Several recent studies showed that many TFs from both eukaryotes and prokaryotes contain additional domains with distinct functions [5,29]. In support of this, a large number of TFs were found to possess at least one other domain besides DBDs among all the putative TFs in cyanobacteria (Fig. 3). These additional domains imply that these TFs might have other functions, which, in some cases, were actually verified by the experimental approaches [5,30]. Several TFs carry binding domains for various substrates or ligands, such as cNMP_binding (cyclic nucleotide-binding domain), LysR_substrate (LysR substrate binding domain), Ada_Zn_binding (metal binding domain of Ada), Fer4 (iron-sulfur cluster binding domain), PAS and GAF. Several TFs encompass enzyme domains with predicted catalytic roles, for example, Aminotran_1_2 (aminotransferase class I and II), GGDEF (diguanylate cyclase activity), HATPase_c (histidine kinase-like ATPases), HisKA (histidine kinase domain), Peptidase_S24 (peptidase family S24), Hpt (histidine-containing phosphotransfer domain) and CbiC (precorrin-8X methylmutases). Otherwise, two kinds of repetitive domains (WD40 and TPR), which are generally responsible for protein-protein interactions, were also found to be associated with DBDs. It this study, however, we also observation that nearly all of the uncommon domain organizations were identified in the genomes of the fresh water or soil cyanobacteria. Although it still needs further proof, our observation suggests that the cyanobacteria living in fresh water or soil tend to have more complex domain organizations of TFs in comparison with those in marine species. The GntR family is an example for this case. The most typical characteristic of this family is that it consists of a single DBD named GntR at N-terminal. In fresh water or soil cyanobacteria, GntR is also fused to several other domains, such as aminotran_1_2, UTRA and FCD. Furthermore, a number of putative TFs (in AraC, CopG, GerE, OmpR or Crp families) with uncommon domain combinations that just appeared in fresh water or soil cyanobacteria are fused to domains involved in signal transduction (including PAS, GAF, Hpt, GGDEF HisKA and HATPase_c). One possible explanation could be that these phenomena are likely to be the results of the fresh water or soil cyanobacteria's efficient response to the physiological and environmental changes during long-term evolution.

Furthermore, we found that there were 12 putative TF families were present in all cyanobacterial genomes. Among them, four families (BolA, DUF387, SfsA and DnaA) have nearly the same gene copies over the genomes, which highlight the fundamental importance of these families. They are presumably very ancient families shared by the most recent common ancestor of cyanobacteria and may have not undergone lineage-specific expansions/loss or horizontal gene transfer. The remaining eight families (OmpR, GerE, Crp, LysR, arsR, FUR, GntR, Bac_DNA_binding) exhibit different distribution patterns among various species. However, we found that a variety of orthologous TFs in these families formed monophyletic clades, which were strongly supported by their high bootstrap values (nearly 100% in several clades) of the constructed phylogenetic trees mentioned above (phylogenetic trees could be queried on the website). Within the families of FUR, Crp, LysR and GntR, only one such branch is found, whereas two branches are observed in GerE and OmpR phylogenies, respectively. ArsR and Bac_DNA_binding phylogenies, however, do not have any such branches. Previously, Brune et al. [2] and Moreno-Campuzano et al. [31] identified the conserved TFs among Corynebacterium and Firmicutes, respectively. Here, we for the first time defined a minimal core of conserved TFs in cyanobacteria: the putative TFs in these nine branches plus these four TF families mentioned above (BolA, DUF387, SfsA and DnaA). These "universal" putative TFs mediate the functions of response regulators of two-component systems (OmpR, GerE), global nitrogen control (Crp), cell-cycle regulation (BolA), sugar fermentation (SfsA), chromosomal replication initiating and regulating (DnaA), general metabolism (GntR), chromosome condensation and segregation (DUF387), iron homeostasis control (FUR), CO2 fixation (LysR) and so on. As the physiological function of most TFs in cyanobacteria is still unknown, this identified core set of conserved TFs might thus provide some guidance for further investigations.

## Conclusion

Currently, the cTFbase is limited to 21 cyanobacterial genomes retrieved from IMG database and works as a centralized warehouse for the comparative genomic analysis of putative TFs in cyanobacteria. Without regular update, however, the database would quickly lose its advantages. Therefore, we prepared to update its data on a regular basis and our update policy is mainly based on following three cases. First, the repertoire of TFs will be identified and integrated into the database when newly completed or draft cyanobacterial genomes are available. Second, novel TFs verified by experiments will be also added into the cTFbase. We encourage users to submit new TFs to our database through the interactive web interface. Third, cTFbase will be updated periodically according to main databases, such as the Pfam and SUPERFAMILY database. Any questions, comments and suggestions will be welcome, which will be a useful feedback for future updating.

## Availability and requirements

Project name

cTFbase: a database for comparative genomics of transcription factors in cyanobacteria

Project home page



Operating system(s)

For user: Standard WWW browser (Safari, Mozilla and Internet Explorer);

For server: Linux

Programming language

PHP, SQL, Perl and Bioperl

License

GNU GPL

Any restrictions to use by non-academics

None

## Authors' contributions

JW performed bioinformatic analysis, constructed the database, developed the web interface, and wrote the manuscript. SW helped with the design of web interface and update of the database. GD, JB and JW prepared the figures in manuscript and website. JQ and JL provided scientific suggestions and criticisms for improving the manuscript and website. QB and FZ participated in its design, helped write the manuscript and supervised the whole project. All authors read and approved the final manuscript.
